# Identifying human postural dynamics and control from unperturbed balance

**DOI:** 10.1186/s12984-021-00843-1

**Published:** 2021-03-22

**Authors:** Jongwoo Lee, Kuangen Zhang, Neville Hogan

**Affiliations:** 1 Department of Mechanical Engineering, Massachusetts Institute of Technology, Cambridge, MA 02139 USA; 2 Department of Mechanical and Energy Engineering, Southern University of Science and Technology, Shenzhen, Guangdong 518055 China; 3 Department of Mechanical Engineering, The University of British Columbia, Vancouver, BC V6T 1Z4 Canada; 4 Department of Brain and Cognitive Sciences, Massachusetts Institute of Technology, Cambridge, MA 02139 USA

**Keywords:** Unperturbed balance, Human quiet standing, System identification, Postural dynamics and control

## Abstract

**Background:**

Upright standing requires control of an inherently unstable multi-joint human body within a small base of support, despite biological motor and / or sensory noise which challenge balance. Without applying perturbations, system identification methods have been regarded as inadequate, because the relevant internal biological noise processes are not accessible to direct measurement. As a result, unperturbed balance studies have been limited to investigation of behavioral patterns rather than possible underlying control strategies.

**Methods:**

In this paper, we present a mathemathically rigorous system identification method that is applicable to study the dynamics and control of unperturbed balance. The method is derived from autocorrelation matrices with non-zero time lags and identifies the system matrix of a discrete-time dynamic system in the presence of unknown noise processes, without requiring any information about the strength of the noise.

**Results:**

Unlike reasonable ‘least-squares’ approaches, the performance of the new method is consistent across a range of different combinations of internal and measurement noise strengths, even when measurement noise is substantial. We present a numerical example of a model that simulates human upright balancing and show that its dynamics can be identified accurately. With a biomechanically reasonable choice of state and input variables, a state feedback controller can also be identified.

**Conclusions:**

This study provides a new method to correctly identify the dynamics of human standing without the need for known external perturbations. The method was numerically validated using simulation that included realistic features of human balance. This method avoids potential issues of adaptation or possible reflex responses evoked by external perturbations, and does not require expensive in-lab, high-precision measurement equipment. It may eventually enable diagnosis and treatment of individuals with impaired balance, and the development of safe and effective assistive and / or rehabilitative technologies.

## Background

### The importance of identifying human postural control

Balance is a fundamental necessity for human mobility. While standing on the ground seems a trivial daily activity, it actually requires sophisticated coordination of the multi-joint human body which is inherently unstable. When humans balance, neural systems for sensory integration, multi-joint coordination, environmental adaptation, and other functions dynamically interact with biomechanical constraints of the musculo-skeletal system [1]. How the central nervous system regulates interaction between neural processes and biomechanics can be better understood by identifying the dynamics of the neural controller that executes corrective joint torques in response to body sway [1–4].

### Previous studies to identify balance

#### Identifying dynamics by perturbing balance

Studies of human postural control can be broadly classified into two different experimental paradigms: perturbed balance and unperturbed balance [4, 5]. In perturbed balance, external perturbations are applied to challenge participants’ balance, e.g. by applying pushing/pulling forces or translating/rotating a platform on which they stand. Those perturbations have traditionally been regarded as necessary to identify the dynamics of human postural control, because the input (external perturbation) and output (motion in response to the perturbation) are directly measured, allowing application of well-established closed-loop system identification techniques to obtain a robust and reliable input-output dynamic relation [2–4, 6]. While insights into sensorimotor control of balance may be gained in this way, it should be noted that humans are notoriously adaptive and are likely to change behavior in response to the applied perturbations [5]. For example, Park et al. [7] showed that postural feedback gains scaled with the magnitude of the applied disturbance. Hence, the closed-loop dynamics and control estimated in this way may not well represent those of daily activity.

#### Understanding natural balance without perturbations

In contrast, unperturbed balance studies do not apply external perturbation. Instead, the only challenges to individuals’ balance arise from internal biological noise in motor and / or sensory systems. The response to this biological noise may be used to investigate humans’ natural postural control. Unperturbed balance also includes studies to understand humans’ remarkable balance ability in challenging environments, such as on a narrow beam [8–11]. In these environments, applying external perturbation is often avoided because the environment itself is so challenging that participants may lose balance before enough data has been collected.

Consistent behavioral patterns observed across individuals, represented by descriptive measures such as center of mass (COM) or center of pressure (COP) motion, suggest strategies to manage complex whole-body balancing in a coordinated manner [8–10, 12–14]. While there is no doubt that characterizing behavioral patterns is important, it does not define the postural control strategy [1]. Identifying the controller solely from behavioral features is quite difficult since different controllers may reproduce the same features observed in experiments [10]. On the other hand, it is quite difficult to apply the system identification techniques which have been widely-employed for perturbed balancing, because the inputs to the system (biological noise) that induce output motion (e.g., sway) in unperturbed balance are *internal* and inaccessible to direct measurement [4]. A reliable system identification method for unperturbed balance would be highly desirable.

### Existing methods

Recently, Ahn and Hogan [15] and Ahn et al. [16] have shown that it is possible to estimate parameters of a noisy, scalar (first-order) dynamical system without external perturbation. Noting that a time series of the dynamical system output can be represented as an autoregressive model of order one, they quantified the bias in estimation based on conventional linear regression methods, then proposed how to compensate for it. Equipped with this revised method, they assessed the gait stability of a model that simulated human walking [15] and, using experimental data, estimated the error-correction gain of a model of human motor learning [16]. Other more classical theories relevant to linear, stationary, white stochastic processes with unknown noise strength have also treated multi-dimensional system parameter identification [17–20].

### Main contribution

The main contribution of this paper is to develop and validate a systematic method to identify the closed-loop dynamics of a multi-joint model of unperturbed human balancing. We formulate this problem as identifying a stochastically-excited, linear, finite-dimensional, discrete-time dynamic system. We exploit auto-correlation matrices of the measurements with non-zero time lags to estimate the parameters of the model. The strengths of the noise processes are not required, which is especially important when identifying unperturbed balance which is driven by unknown internal noise. To better understand the key properties of the new method, we first consider a simple scalar dynamic model. Then we present a numerical example of a model that simulates human upright balancing and show that its dynamics can be identified accurately. Assuming the dynamic structure of a stochastically-excited double-inverted-pendulum model, a state feedback controller can also be identified. Conversely, comparable parameters estimated using conventional least squares methods exhibit large errors.

The method proposed in this paper is largely inspired by similar approaches developed to identify human gait stability [15], human motor learning dynamics [16], and brain activity from electroencelphalogram (EEG) signals [20]. While those studies did not consider measurement noise separately from biological noise, we show that measurement noise can cause significant bias in estimation. We also present a way to mitigate the problem. Equipped with the new method, *natural* human postural dynamics and control can be studied in depth without concern for adaptation or possible reflex responses evoked by external perturbations, or any need for expensive high-precision measurement equipment. Reliable quantitative identification of the dynamics and control of human balance, as presented in this paper, would enable diagnosis and treatment of individuals with impaired balance, and the development of safe and effective assistive and / or rehabilitative technologies.

## Methods

### Identifying a general system from autocorrelation matrices

Consider a discrete-time stochastic finite-dimensional linear time-invariant dynamic system

1 $$\begin{aligned} {\left\{ \begin{array}{ll} {\mathbf {x}}_{t+1}=\mathbf {Ax}_t+\mathbf {Gw}_t\\ {\mathbf {z}}_{t}=\mathbf {Hx}_t+{\mathbf {v}}_t, \end{array}\right. } \end{aligned}$$

where ${\mathbf {x}}\in \mathbb {R}^{n_x}, {\mathbf {z}}\in \mathbb {R}^{n_z}$ are state and measured output vectors, respectively, at time *t*. We assume that process noise, ${\mathbf {w}}\in \mathbb {R}^{n_w}$, and measurement noise, ${\mathbf {v}}\in \mathbb {R}^{n_v}$, are white and uncorrelated:

$$\begin{aligned} E\{{\mathbf {w}}_t\}={\mathbf {0}}, \quad&E\{{\mathbf {w}}_t{\mathbf {w}}_s^T\}={\varvec{\Sigma }}_{{\mathbf {w}}}\delta _{ts}\\ E\{{\mathbf {v}}_t\}={\mathbf {0}},\quad&E\{{\mathbf {v}}_t{\mathbf {v}}_s^T\}={\varvec{\Sigma }}_{{\mathbf {v}}}\delta _{ts}\\ E\{{\mathbf {w}}_t{\mathbf {v}}_s^T\}={\mathbf {0}}\quad&\forall t, s \end{aligned}$$

The objective is to estimate the $n_x\times n_x$ system matrix ${\mathbf {A}}$. We first compute the auto-correlation matrix of the output ${\mathbf {z}}$ with non-zero lag $k>0$ as

2 $$\begin{aligned}&{\mathbf {R}}_{\mathbf {zz}}(k)\\&=E\{ {\mathbf {z}}_t {\mathbf {z}}_{t-k}^T \}\\&=E\{ (\mathbf {Hx}_t+{\mathbf {v}}_t)(\mathbf {Hx}_{t-k}+{\mathbf {v}}_{t-k})^T \}\\&=E\{\mathbf {Hx}_t{\mathbf {x}}_{t-k}^T {\mathbf {H}}^T+\mathbf {Hx}_t {\mathbf {v}}_{t-k}^T +{\mathbf {v}}_t{\mathbf {x}}_{t-k}^T{\mathbf {H}}^T+ {\mathbf {v}}_t {\mathbf {v}}_{t-k}^T \}\\&={\mathbf {H}}E\{{\mathbf {x}}_t{\mathbf {x}}_{t-k}^T \}{\mathbf {H}}^T = \mathbf {HR}_{\mathbf {xx}}(k){\mathbf {H}}^T \end{aligned}$$

where ${\mathbf {R}}_{\mathbf {zz}}(0)$ can be obtained as

3 $$\begin{aligned} &{\mathbf {R}}_{\mathbf {zz}}(0)\\&=E\{ {\mathbf {z}}_t {\mathbf {z}}_{t}^T \}\\&=E\{ (\mathbf {Hx}_t+{\mathbf {v}}_t)(\mathbf {Hx}_{t}+{\mathbf {v}}_{t})^T \}\\&=E\{\mathbf {Hx}_t{\mathbf {x}}_{t}^T {\mathbf {H}}^T+\mathbf {Hx}_t {\mathbf {v}}_{t}^T +{\mathbf {v}}_t{\mathbf {x}}_{t}^T{\mathbf {H}}^T+ {\mathbf {v}}_t {\mathbf {v}}_{t}^T \}\\&={\mathbf {H}}E\{{\mathbf {x}}_t{\mathbf {x}}_{t}^T \}{\mathbf {H}}^T + E\{ {\mathbf {v}}_t {\mathbf {v}}_{t}^T \}= \mathbf {HR}_{\mathbf {xx}}(0){\mathbf {H}}^T + {\varvec{\Sigma }}_{\mathbf {v}} \end{aligned}$$

An expression for ${\mathbf {R}}_{\mathbf {xx}}(k)$ for the dynamic system (1) can easily be obtained. Noting that $E\{{\mathbf {x}}_t{\mathbf {w}}_s^T\} ={\mathbf {0}}$ for $t \le s$,

4 $$\begin{aligned} {\mathbf {R}}_{\mathbf {xx}}(k)&=E\{ {\mathbf {x}}_t {\mathbf {x}}_{t-k}^T \}\\&=E\{ ({\mathbf {A}}^k{\mathbf {x}}_{t-k}+\sum _{j=1}^{k} {\mathbf {A}}^{j-1}\mathbf {Gw}_{t-j}){\mathbf {x}}_{t-k}^T \}\\&={\mathbf {A}}^kE\{{\mathbf {x}}_{t-k}{\mathbf {x}}_{t-k}^T\}={\mathbf {A}}^k{\mathbf {R}}_{\mathbf {xx}}(0) \end{aligned}$$

where ${\mathbf {R}}_{\mathbf {xx}}(0)$ can be obtained as

5 $$\begin{aligned} {\mathbf {R}}_{\mathbf {xx}}(0)&= {\mathbf {P}} =E\{ {\mathbf {x}}_t {\mathbf {x}}_{t}^T \}\\&=E\{ (\mathbf {Ax}_{t-1}+\mathbf {Gw}_{t-1})(\mathbf {Ax}_{t-1}+\mathbf {Gw}_{t-1})^T \}\\&=E\{ \mathbf {Ax}_{t-1}{\mathbf {x}}_{t-1}^T{\mathbf {A}}^T+\mathbf {Gw}_{t-1}{\mathbf {w}}_{t-1}^T{\mathbf {G}}^T \}\\&=\mathbf {AR_{xx}}(0){\mathbf {A}}^T + {\mathbf {G}}{\varvec{\Sigma }}_\mathbf {wG}^T \\&=\mathbf {APA}^T + {\mathbf {G}}{\varvec{\Sigma }}_\mathbf {wG}^T \end{aligned}$$

From (2) and (4), it readily follows that

6 $$\begin{aligned} {\mathbf {R}}_{\mathbf {zz}}(k) = \mathbf {HA}^k\mathbf {PH}^T, \forall k>0 \end{aligned}$$

If ${\mathbf {H}}^{-1}$ exists, one can derive the matrix *A* from autocorrelation matrices as

7 $$\begin{aligned} {\mathbf {A}} = {\mathbf {H}}^{-1}{\mathbf {R}}_{\mathbf {zz}}(k+1){\mathbf {R}}_{\mathbf {zz}}(k)^{-1}{\mathbf {H}}, \end{aligned}$$

Note that (7) holds for all $k>0$.

We now turn to the estimation problem. Using the ergodic property of ${\mathbf {z}}_t$, ${\mathbf {R}}_{\mathbf {zz}}(k)$ can be estimated as $\frac{1}{N-k}\sum _{t=k+1}^N {\mathbf {z}}_t {\mathbf {z}}_{t-k}^T$ for $k \ge 0$, where *N* is the length of the time series. As long as the process is ergodic, it has been shown that ${\hat{\mathbf {R}}}_\mathbf {zz}(k)$ provides an asymptotically unbiased, normal, and consistent estimate [21]. The estimation can be improved by either increasing the trial duration (N) or combining multiple-trial data of each participant. In practice, the trial duration cannot be arbitrarily extended because participants’ dynamics may vary over time due to fatigue. Denoting $n_{T}$ as the total number of trials per participant and ${\hat{\mathbf {R}}}_\mathbf {zz}^{(i)}(k)$ as the estimated autocorrelation matrix for *i*-th trial, we can re-define ${\hat{\mathbf {R}}}_\mathbf {zz}(k)$ as

$$\begin{aligned} {\hat{\mathbf {R}}}_{\mathbf {zz}}(k)&= \frac{1}{n_T} \sum _{i=1}^{n_T} {\hat{\mathbf {R}}}_{\mathbf {zz}}^{(i)}(k) \\&= \frac{1}{n_T}\frac{1}{N-k}\sum _{i=1}^{n_T}\sum _{t=k+1}^N {\mathbf {z}}^{(i)}_t {\mathbf {z}}^{(i)T}_{t-k}. \end{aligned}$$

where ${\mathbf {z}}^{(i)}$ is the measured output of *i*-th trial. From (7), we obtain an expression for the estimate of ${\mathbf {A}}$ as

8 $$\begin{aligned} {\hat{\mathbf {A}}}_{\mathrm{CR}} = {\mathbf {H}}^{-1}{\hat{\mathbf {R}}}_\mathbf {zz}(k+1){\hat{\mathbf {R}}}_\mathbf {zz}(k)^{-1}{\mathbf {H}}, \forall k>0, \end{aligned}$$

where the subscript CR stands for correlation.

In practice, since ${\mathbf {R}}_{\mathbf {zz}}(k)$  $=\mathbf {HR}_{\mathbf {xx}}(k){\mathbf {H}}^T$  $=\mathbf {HA}^k{\mathbf {R}}_{\mathbf {xx}}(0){\mathbf {H}}^T$, for a stable system with $\Vert {\mathbf {A}}\Vert <1$, too large a value of *k* will cause ${\mathbf {R}}_{\mathbf {zz}}(k)$ to have a large condition number, which may amplify numerical error and degrade the quality of estimate. To alleviate this performance degradation, by noting that the relation ${\mathbf {A}}{\mathbf {H}}^{-1}{\mathbf {R}}_{\mathbf {zz}}(k)={\mathbf {H}}^{-1}{\mathbf {R}}_{\mathbf {zz}}(k+1)$ holds for all $k>0$, (8) can be improved

9 $$\begin{aligned} {\hat{\mathbf {A}}}_{\mathrm{CR}(m)}=&{\mathbf {H}}^{-1}[{\hat{\mathbf {R}}}_\mathbf {zz}(2), \ldots, {\hat{\mathbf {R}}}_\mathbf {zz}(m+1)]\\&\times [{\hat{\mathbf {R}}}_\mathbf {zz}(1), \ldots, {\hat{\mathbf {R}}}_\mathbf {zz}(m)]^+{\mathbf {H}} \end{aligned}$$

for a hyperparameter *m*, where $\cdot ^+$ denotes a pseudo-inverse operator. We will briefly discuss the properties of the estimation methods in the **Results** Section.

### Identifying controller gain

In order to apply (9) to identify human postural control, consider a controllable system

10 $$\begin{aligned} {\left\{ \begin{array}{ll} {\mathbf {x}}_{t+1}=\mathbf {Ax}_t+\mathbf {Bu}_t+\mathbf {Gw}_t\\ {\mathbf {z}}_{t}=\mathbf {Hx}_t+{\mathbf {v}}_t \end{array}\right. }, \end{aligned}$$

where ${\mathbf {u}}\in \mathbb {R}^{n_u}$ is control input and ${\mathbf {B}}$ is input weighting matrix. If a balancing human is modeled as a set of kinematically coupled rigid segments, with an appropriate choice of generalized coordinates the structure of ${\mathbf {A}}$ and ${\mathbf {B}}$ may be determined from equations of motion using standard methods. For example, if relative joint angles and angular velocities are chosen as the state vector ${\mathbf {x}}$ and joint torques as the input vector ${\mathbf {u}}$, the system matrix ${\mathbf {A}}$ and input matrix ${\mathbf {B}}$ are constrained by the dynamic structure. In particular, if joint angles comprise the first elements of ${\mathbf {x}}$, ${\mathbf {B}}$ must have $[{\mathbf {0}}]$ as its top $n_x/2$ rows. The corresponding rows of ${\mathbf {A}}$ have a a unity block $[{\mathbf {I}}]$ in the first $n_x/2$ columns. The second half of the matrix is determined by the continuous-to-discrete time conversion rule and sampling frequency, as the first rows of the corresponding matrix in continuous-time consist of a $[{\mathbf {0}}]$ block and a unity block $[{\mathbf {I}}]$; see Appendix 4: Discrete-to-continuous conversion. The dimensions and precise meaning of the rest of ${\mathbf {A}}$ and ${\mathbf {B}}$ depend on the system configuration, state vector, and control input. For instance, modeling a human as a planar inverted pendulum with two joints (ankle and hip), one may assume the pendulum is controlled either by joint torque actuators (${\mathbf {u}}$: joint torques) or muscle actuators (${\mathbf {u}}$: muscle forces), depending on the purpose of the model. While these assumptions may be restrictive, they are biomechanically reasonable and establish the structure of ${\mathbf {A}}$ and ${\mathbf {B}}$.

Next, suppose the system is equipped with a feedback controller that stabilizes the system about its operating point $\mathbf {x=0}$,

11 $$\begin{aligned} {\left\{ \begin{array}{ll} {\mathbf {y}}_{t}=\mathbf {Cx}_t+\mathbf {Du}_t+{\mathbf {e}}_t\\ {\mathbf {u}}_t = -\mathbf {Ky}_t+\varvec{\eta }_t \end{array}\right. } \end{aligned}$$

where ${\mathbf {y}}\in \mathbb {R}^{n_y},{\mathbf {e}}\in \mathbb {R}^{n_e}, \varvec{\eta }\in \mathbb {R}^{n_\eta }$ are sensory signals fed back to a stabilizing controller, sensory noise, and motor noise, respectively. ${\mathbf {K}}$ is the $n_u \times n_y$ gain matrix. Without loss of generality and for simplicity, we can assume $\mathbf {D=0}$ (Extension of the method to non-zero ${\mathbf {D}}$ would be straightforward, but is left for future work.). The closed-loop system equipped with the controller (10)-(11) is reduced to

12 $$\begin{aligned} {\left\{ \begin{array}{ll} {\mathbf {x}}_{t+1}={\mathbf {A}}_\mathbf {cl}{\mathbf {x}}_{\mathbf {t}}+{\mathbf {G}}_{cl}\tilde{{\mathbf {w}}}_t\\ {\mathbf {z}}_{t}=\mathbf {Hx}_t+{\mathbf {v}}_t \end{array}\right. } \end{aligned}$$

where ${\mathbf {A}}_\mathbf {cl}={\mathbf {A}}-\mathbf {BKC}$,   ${\mathbf {G}}_{\mathbf {cl}} = \mathbf{[G, -BK, B]}$, and $\tilde{\mathbf{w}}=[\mathbf{w}^T, \mathbf{e}^T, \mathbf{\varvec{\eta }}^T]$. We assume that the noise sources ${\mathbf {w}}, {\mathbf {e}}$, and $\varvec{\eta }$ are white, mutually uncorrelated, and with covariance matrices ${{\varvec{\Sigma }}_{\mathbf {w}}, {\varvec{\Sigma }}_{\mathbf {e}}, {\varvec{\Sigma }}_{\varvec{\eta }}}$, respectively. An asymptotically unbiased and consistent estimate ${\hat{\mathbf {A}}}_\mathbf {cl}$ can be obtained using the procedure of (9).

One can further estimate the gain matrix ${\mathbf {K}}_{\mathbf {x}} = \mathbf {KC}$ of the state-feedback controller (11) by solving the following linear regression problem,

13 $$\begin{aligned} {\hat{\mathbf {K}}}_\mathbf {x }= \mathbf{B}^+({\mathbf {A}}-{\hat{\mathbf {A}}}_\mathbf {cl}) \end{aligned}$$

Note that for $n_u<n_x$, this is an over-determined problem and its unique solution can be obtained. Note also that the controller gain can be estimated in the continuous-time domain using a proper discrete-to-continuous time model conversion, as described in Appendix 4: Discrete-to-continuous conversion.

This method requires *a priori* knowledge of ${\mathbf {A}}$ and ${\mathbf {B}}$ but those are determined by the mechanical physics of the model assumed to describe experimental human balancing data. Note especially that if joint angles and angular velocities are chosen as the state vector ${\mathbf {x}}$ and joint torques (with zero mechanical impedance) as the input vector ${\mathbf {u}}$, constructing ${\mathbf {A}}$ and ${\mathbf {B}}$ for the open-loop (uncontrolled) system only requires knowledge of kinematics and gravito-inertial mechanics. Geometric and inertial properties of limb segments are quite well quantified in the literature, for example see [22]. In this way, the method presented here ‘fills in’ the missing data about mechanical impedance.

## Results

### Numerical simulation: scalar dynamic system

#### Model

To gain insight, consider a simple stable dynamic system,

14 $$\begin{aligned} {\left\{ \begin{array}{ll} x_{t+1} = ax_t+gw_t\\ z_t = hx_t + v_t, \end{array}\right. } \end{aligned}$$

where *a*, *g*, *h* are unknown scalar system parameters. Unknown noise processes are drawn from zero-mean Gaussian distributions, $w_t \sim {\mathcal {N}}(0, \sigma _w^2), v_t \sim {\mathcal {N}}(0, \sigma _v^2)$. We assume $|a|<1$, i.e., the system is stable. It can readily be obtained from (2) - (5) that

$$\begin{aligned} {\left\{ \begin{array}{ll} R_{xx}(0)=P=a^2P+g^2\sigma _w^2 = \frac{1}{1-a^2}g^2\sigma _w^2 \\ R_{xx}(k) = a^kP, \forall k\ge 0\\ R_{zz}(0) = h^2P+\sigma _v^2 = \frac{1}{1-a^2}g^2h^2\sigma _w^2+\sigma _v^2\\ R_{zz}(1) = ah^2P\\ R_{zz}(k+1) = aR_{zz}(k), \forall k \ge 1 \end{array}\right. } \end{aligned}$$

#### Simulation setup

For this simple system, we compared the new method (9), ${\hat{a}}_{\mathrm{CR(m)}}$ with different *m*-values ($m=1$ and $m=10$), with the ordinary least-squares method (OLS), ${\hat{a}}_{\mathrm{OLS}}$. The ordinary least-squares method is detailed in Appendix 1: Ordinary least squares; note that the estimate yielded by OLS is equivalent to that by the Yule-Walker equations, which are widely used [15, 20]. In the following numerical example, we simulated the dynamic system (14) with $h=g=1$ for different system parameters $a \in (-1, 1)$ with a finite resolution of 0.1. The estimates ${\hat{a}}_{\mathrm{CR(m)}}$ and ${\hat{a}}_{\mathrm{OLS}}$ were computed from five different trials ($n_T=5$) and each trial consisted of a time series with length $N=3000$. This corresponds to 30s of simulation with a sampling rate of 100Hz, typical for studies of human behavior. The noise strengths $\sigma _w, \sigma _v$ were also varied such that the relative strength $\sigma _r=\sigma _v/\sigma _w$ was 0, 1/2, 1, and 2. Finally, to understand the statistical properties of the estimation methods, we iterated the above procedure 100 times and obtained the mean and standard deviation of the error of estimation, ${\hat{a}}_{(\cdot )}-a$. All simulations and computations were conducted in MATLAB 2018b (Mathworks, MA).

#### Simulation result

**Fig. 1:**
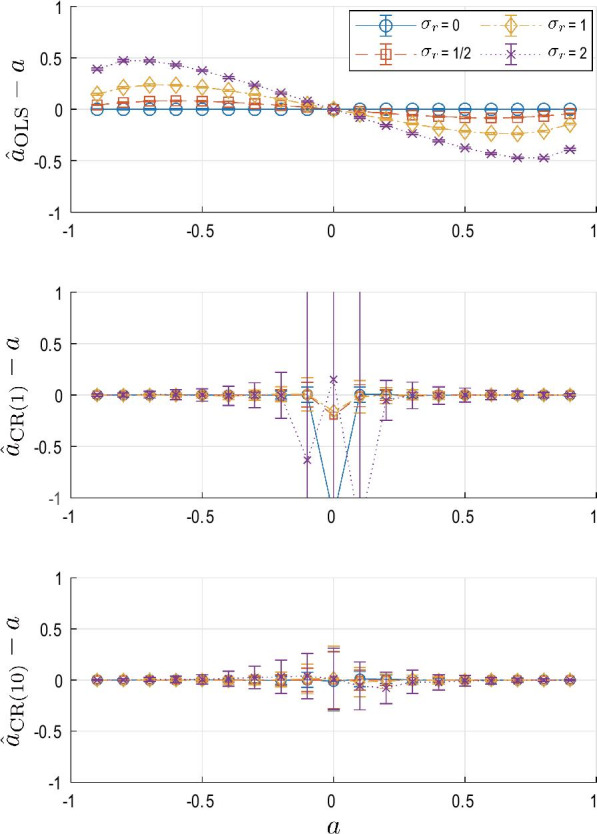
Comparison of estimation methods with different process and measurement noise strengths. Each estimate was obtained from 5 different trials. Each trial consisted of a time series with length N = 3000. The mean and standard deviation of the error of estimation (${\hat{a}}-a$) for each plot were obtained from 100 iterations of the whole process

Figure 1 compares the performance of different estimation methods. The ordinary least-squares estimate ${\hat{a}}_{\mathrm{OLS}}$ shows small variance but non-zero bias. The mean error of the estimate is zero at $a=0$, but the bias at large |*a*| is considerable and probably unacceptable. On the other hand, ${\hat{a}}_{\mathrm{CR}(m)}$ is not biased when the system parameter *a* is non-zero and large. However, when $|a|\sim 0$, its performance is degraded. In general, the variance and mean error of all methods decrease as relative noise strength $\sigma _r$ increases, i.e., with more accurate measurements and larger internal perturbation.

To understand the difference between ${\hat{a}}_{\mathrm{OLS}}$ and ${\hat{a}}_{\mathrm{CR}(1)}$, it is convenient to derive analytic expressions. The ordinary least squares method is given as

15 $$\begin{aligned} {\hat{a}}_{\mathrm{OLS}} = \frac{{\hat{R}}_{zz}(1)}{{\hat{R}}_{zz}(0)} \approx \frac{R_{zz}(1)}{R_{zz}(0)} \end{aligned}$$

and ${\hat{a}}_{\mathrm{CR}(1)}$ is given as

16 $$\begin{aligned} {\hat{a}}_{\mathrm{CR}(1)} = \frac{{\hat{R}}_{zz}(2)}{{\hat{R}}_{zz}(1)} \approx \frac{R_{zz}(2)}{R_{zz}(1)} \end{aligned}$$

where

17 $$\begin{aligned}&\frac{R_{zz}(1)}{R_{zz}(0)} =\frac{ah^2P}{h^2P+\sigma _v^2}=\frac{a}{1+(1-a^2)\frac{\sigma _v^2}{{h^2g^2\sigma }_w^2}} \end{aligned}$$

18 $$\begin{aligned}&\frac{R_{zz}\left( 2\right) }{R_{zz}\left( 1\right) }=\frac{a^2h^2P}{ah^2P}=a \end{aligned}$$

It is clear that even if autocorrelation is perfectly estimated, e.g., ${\hat{R}}_{zz}(k)=R_{zz}(k)$, ${\hat{a}}_{\mathrm{OLS}}$ has bias which depends on both the system parameters *a*, *g*, *h* and the unknown noise strengths $\sigma _w^2, \sigma _v^2$, while ${\hat{a}}_{\mathrm{CR}(1)}$ provides an unbiased estimate without requiring any information about the noise strengths. In particular, the bias in ${\hat{a}}_{\mathrm{OLS}}$ increases as the relative noise $\sigma _v/\sigma _w$ increases. On the other hand, ${\hat{a}}_{\mathrm{CR}(1)}$ is not well defined for $|a|\sim 0$ because its denominator contains *a*. These properties are well represented in Fig. 1. While ${\hat{a}}_{\mathrm{OLS}}$ has smaller variance for all *a* values, the error due to bias is substantial for non-zero *a*. ${\hat{a}}_{\mathrm{CR}(1)}$ has relatively large variance in general but provides quite an accurate estimate unless *a* is close to 0. When true *a* is close to 0, ${\hat{a}}_{\mathrm{CR}(1)}$ is quite imprecise.

This drawback can be overcome if we use ${\hat{a}}_{\mathrm{CR}(10)}$. This estimate for large true *a* is as accurate and exhibits as little bias as ${\hat{a}}_{\mathrm{CR}(1)}$. More importantly, it is remarkable that ${\hat{a}}_{\mathrm{CR}(10)}$ substantially improves accuracy and variance even when $|a|\sim 0$. While its variance is still larger than ${\hat{a}}_{\mathrm{OLS}}$, the accuracy of its mean value is comparable.

**Fig. 2:**
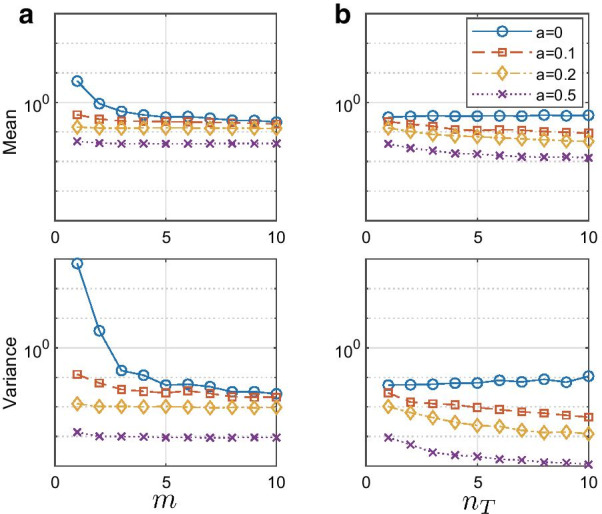
The effect of hyper-parameters *m*, the maximum time lag of the autocorrelation function used to estimate ${\hat{a}}$, and $n_T$, the total number of trials, on $|{\hat{a}}_{\mathrm{CR(m)}}-a|$. Noise strengths were fixed as $\sigma _w=\sigma _v=1$. **A** $n_T = 5$ was fixed and *m* was varied. **B** $m=5$ was fixed and $n_T$ was varied

We also tested the effect of hyper-parameters *m*, the maximum time lag in autocorrelation to estimate ${\hat{a}}$, and $n_T$, the total number of trials, on the error of estimation and present the result in Fig. 2. The absolute value of the error of estimation, $|{\hat{a}}_{\mathrm{CR(m)}}-a|$ was computed, then the average and variance of the absolute error were computed from 100 iterations. As shown in Fig. 2, in general both hyper-parameters monotonically improved the reliability of estimation by reducing both mean error and its variance. As might be expected, increasing the number of trials had more effect than *m*. This is because increasing *m* means more ${\hat{R}}_{zz}(k)$ are recruited for ${\hat{a}}$, while increasing the number of trials helps to better estimate $R_{zz}(k)$ and consequently reduces the errors that propagate in estimating ${\hat{a}}$. Thus it is always recommended to use as large as $n_T$ as possible, i.e., collect as many data as possible from each participant.

The performance improvement with increasing *m* quickly reached a plateau, and thus a sufficiently large value of *m*, for instance $m = 10$ can be chosen to improve the estimation. As can be seen in Figs. 1 and  2, the variance when $|a|\sim 0$ is still quite large. Therefore one may first compute ${\hat{a}}_{\mathrm{OLS}}$ to estimate *a*, then compute ${\hat{a}}_{\mathrm{CR(m)}}$ when $|{\hat{a}}_{\mathrm{OLS}}|$ is larger than a threshold, e.g., 0.1. For higher dimensional systems, one may instead use the norms of ${\mathbf {R}}_{\mathbf {zz}}(k)$ and ${\mathbf {R}}_{\mathbf {zz}}(1)$ to determine the value of *m*.

### Numerical simulation: balance model

#### Double inverted pendulum model

**Fig. 3:**
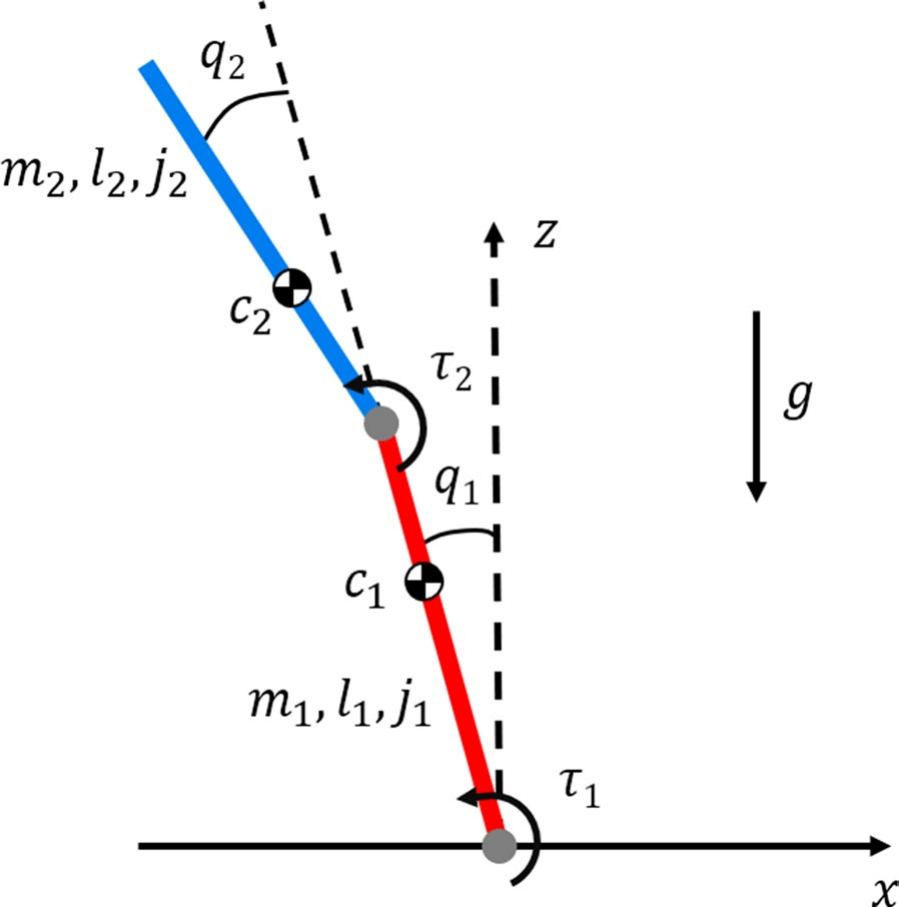
Double inverted pendulum model with angle ($q_i$) and torque ($\tau _i$) sign conventions and parameter values for mass ($m_i$), length ($l_i$), center of mass ($c_i$), and moment of inertia about center of mass ($j_i$). The direction of gravity (*g*) is also defined

Human quiet standing is often modeled as an inverted pendulum with single [23], double [10], or more than two joints [24]. To establish how the new method performs for a multi-joint case, we adopted a double inverted pendulum model of human quiet standing. Lumped model parameters including mass, center of mass position from joint, length, moment of inertia about center of mass of each link, and gravitational acceleration are listed in Table 1. They were computed based on the anthropomorphic distribution of males [22], with weight and height of 73 kg and 1.73 m. We assumed that the foot is not moving during standing and regarded the ankle as a pin joint; any mass and length below the ankle was neglected in the double-inverted pendulum model. Figure 3 illustrates joint angles and torques for ankle ($q_1, \tau _1$) and hip ($q_2, \tau _2$). As in (11), it was assumed that each joint torque is a sum of control input torque and actuation error, $\tau _i = u_i + \eta _i$. The state vector ${\mathbf {x}}=[q_1, q_2, {\dot{q}}_1, {\dot{q}}_2]^T$ and input vector ${\mathbf {u}} = [u_1, u_2]^T$ were defined accordingly.

**Table 1:** double inverted pendulum model parameters

Symbol	Parameter meaning (units)	Value
$m_1$	Mass of link 1 (kg)	25.89
$l_1$	Length of link 1 (m)	0.857
$c_1$	Center of mass of link 1 (m)	0.582
$j_1$	Moment of inertia of link 1 (kgm2)	1.350
$m_2$	Mass of link 2 (kg)	42.20
$l_2$	Length of link 2 (m)	0.841
$c_2$	Center of mass of link 2 (m)	0.328
$j_2$	Moment of inertia of link 2 (kgm$^2$)	2.547
g	Gravitational acceleration (m/s$^2$)	9.81

#### Stabilizing controller

We used an infinite-horizon linear quadratic regulator (LQR) to stabilize the double inverted pendulum. The LQR is a state-feedback controller in which gain ${\mathbf {K}}_{\mathbf {x}}$ is determined such that a quadratic cost is minimized:

$$\begin{aligned} {\mathbf {K}}_{\mathbf {x}} = {{\,\mathrm{\mathrm{argmin}}\,}}_{{\mathbf {K}}_{\mathbf {x}}} \int _0^\infty [{\mathbf {x}}^T(t)\mathbf {Qx}(t)+{\mathbf {u}}^T(t)\mathbf {Ru}(t)] \mathrm {dt}, \end{aligned}$$

where $\mathbf {u=-K_x}\mathbf {x}$ [25]. Two sets of parameters of the LQR were tested:

- Case 1:
  $$\begin{aligned} {\mathbf {Q}}={\mathbf {I}}_4, \quad {\mathbf {R}}= \begin{bmatrix} 5 &{} 0 \\ 0 &{} 1/5 \end{bmatrix}. \end{aligned}$$
- Case 2:
  $$\begin{aligned} {\mathbf {Q}}={\mathbf {I}}_4, \quad {\mathbf {R}}=10^6 \begin{bmatrix} 0.3 &{} 0 \\ 0 &{} 10/3 \end{bmatrix}. \end{aligned}$$

The parameters used in Case 1 are those which were reported as well-representing human balancing and similar to the ‘hip strategy’ [10, 26]. Case 2 was intended to test a different type of controller which encouraged more use of the ‘ankle’, similar to the ‘ankle strategy’, but minimized control effort.

Finally, the torque controller was perturbed by internal sensory noise ${\mathbf {e}}$ and motor noise $\varvec{\eta }$ with ${{\varvec{\Sigma }}_{\mathbf {e}}} = \sigma _e^2{\mathbf {I}}_4$ and ${{\varvec{\Sigma }}_{\varvec{\eta }}} = \sigma _\eta ^2 {\mathbf {I}}_2$ such that

$$\begin{aligned} \varvec{\tau }= {\mathbf {u}}+\varvec{\eta }= -{\mathbf {K_x}}\mathbf {(x+e)}+\varvec{\eta }. \end{aligned}$$

#### Model linearization

While we used the full nonlinear equations of motion to simulate human balance, when stabilized by the LQR and perturbed by small internal noise, the resultant motion of the double inverted pendulum is subtle, consistent with experimental observations of quiet standing [6, 27]. For small motion, the nonlinear system can be well-approximated as a linear system (10), as detailed in Appendix 3: Double inverted pendulum linearization. From the linearized model, ${\mathbf {A}}_{\mathrm{cl}}$ was obtained.

#### Simulation setup

We used the new method to estimate the closed-loop system matrix ${\mathbf {A}}_{\mathrm{cl}}$ and controller gain matrix ${\mathbf {K}}_{\mathbf {x}}$. Because the model was developed in continuous-time, we first estimated discrete-time model parameters using (9), then converted them into continuous-time model parameters by following the method described in Appendix 4: Discrete-to-continuous conversion. The size of the error between true and estimated matrices was computed as below

19 $$\begin{aligned} e_A = (\Vert {\mathbf {A}}_{\mathrm{cl}}-{\hat{\mathbf {A}}}_{\mathrm{cl}} \Vert _2) / \Vert {\mathbf {A}}_{\mathrm{cl}} \Vert _2 \ \end{aligned}$$

20 $$\begin{aligned} e_K = (\Vert {\mathbf {K}}_{\mathbf {x}}-{\hat{\mathbf {K}}}_{\mathbf {x}} \Vert _2) / \Vert {\mathbf {K}}_{\mathbf {x}} \Vert _2 \end{aligned}$$

Note that from the choice of the state vector ${\mathbf {x}}$, the first two rows of ${\mathbf {A}}_{\mathrm{cl}}$ are constrained to $[\mathbf {0, I}]$. Therefore, we replaced the first two rows of ${\hat{\mathbf {A}}}_{\mathrm{cl}}$ with $[\mathbf {0, I}]$ to obtain the controller gain using (13) and compute errors.

Similar to **Scalar Dynamic System** example, the errors obtained from the new method and from the ordinary least squares method were compared for different combinations of noise strengths. Note that sensory and motor noise are essentially equivalent in this setup, e.g., ${\mathbf {u}}_t = \mathbf {-KCx}_t-\mathbf {Ke}_t+\varvec{\eta }_t= \mathbf {-KCx}_t+\tilde{\varvec{\eta }}_t$. Thus, in the following simulation we fixed $\sigma _\eta$ and varied $\sigma _e$. The tested parameters are summarized in Table 2.

**Table 2:** Range of noise strengths tested

Symbol	Parameter meaning	Range
$\sigma _e$	Sensory noise strength	[1e−02, 3e−02]
$\sigma _\eta$	Motor noise strength	1e−02
$\sigma _v$	Measurement noise strength	[1e−03, 5e−03]

${\hat{\mathbf {A}}}_{\mathrm{cl}}$ was computed from five different trials ($n_T=5$). In each trial, a semi-implicit Euler integrator was used to simulate forward dynamics of the model for 90 s with a time step of 0.01 s (100Hz sampling rate, $N=9000$). Finally, in order to understand the statistical properties of each estimation method, we iterated the above procedure 10 times and obtained the mean and standard deviation of $e_{A,(\cdot )}$ and $e_{K, (\cdot )}$. All simulations and computations were conducted in MATLAB 2018b (Mathworks, MA).

#### Results

**Fig. 4:**
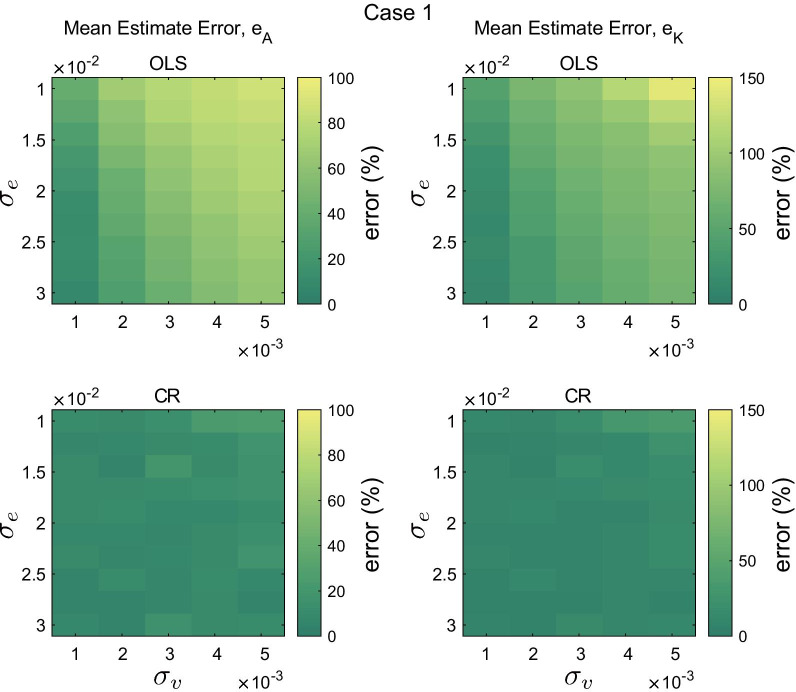
Mean estimated error of the system matrix $e_A$(%, left) and the control gain $e_K$(%, right) from 10 iterations for different noise combinations. Errors of the ordinary least squares method (OLS, top) and the new method (CR, bottom) are shown. For both cases, motor noise was fixed as $\sigma _\eta = 0.01$. The double inverted pendulum model was simulated with the Case 1 controller parameters

**Fig. 5:**
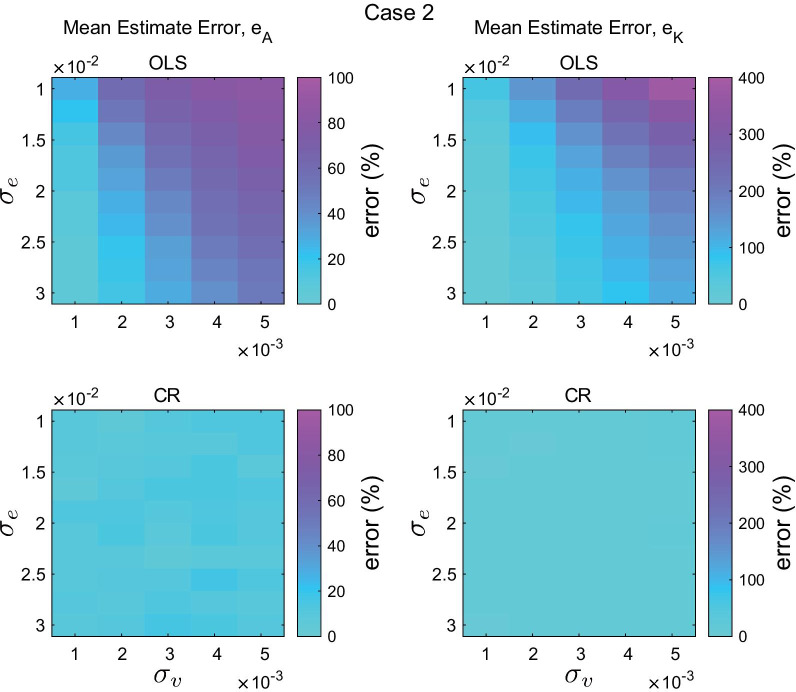
Mean estimated error of the system matrix $e_A$(%, left) and the control gain $e_K$(%, right) from 10 iterations for different noise combinations. Errors of the ordinary least squares method (OLS, top) and the new method (CR, bottom) are shown. For both cases, motor noise was fixed as $\sigma _\eta = 0.01$. The double inverted pendulum model was simulated with the Case 2 controller parameters

Figures 4 and  5 present the mean error of estimation of the system matrix $e_A$ and the controller gain $e_K$ obtained from ${\hat{\mathbf {A}}}_{\mathrm{cl, OLS}}$ and ${\hat{\mathbf {A}}}_{\mathrm{cl, CR}}$ for various combinations of noise strengths and for two different controllers. In general, increasing measurement noise degraded performance estimation. For example, in Case 1, when $\sigma _e = 0.01$ and $\sigma _\eta = 0.01$, the mean $e_{A, \mathrm{OLS}}$ was 40.5% with $\sigma _v = 0.001$ but 86.9% with $\sigma _v = 0.005$. Increasing sensory noise improved the estimate of the ordinary least squares method, yet its performance remained much worse than that obtained from the new method. The performance gap between the ordinary least squares method and the new method was even larger when estimating controller gain. For example, the mean error of estimation $e_K$ from the ordinary least squares method reached about 150%.

Within the range of parameters tested, the error of estimation from the new method was slightly affected by different levels of noise. The mean and standard deviation of the error of estimation for all conditions were about 10% and 9% for $e_A$ and 11% and 10% for $e_K$, respectively.

The performance gap between the ordinary least squares method and the new method was even larger in Case 2, as shown in Fig. 5. For example, the mean error of estimation $e_K$ from the ordinary least squares method reached over 400% (note the scale of the color bar), while the mean and standard deviation of the error of estimation from the new method for all conditions were about 10% and 7% for $e_A$ and 9% and 6% for $e_K$, respectively.

## Discussion

### Summary of the work

In this work, we presented an unbiased parametric system identification method that enables estimating the dynamics of human postural control using recorded joint trajectories without external perturbation. While the physical world is in the continuous-time domain, our digital measurement systems provide us signals in the discrete-time domain. Hence, we investigated a method to identify a discrete-time model. With a biomechanically reasonable model of the multi-joint human body, the gain matrix of a state-feedback controller can also be estimated. We first examined the properties of the new method using a simple scalar dynamic system. While the ordinary least squares method showed bias due to unknown noise in the system, the new method did not show bias even without information about the system’s noise strengths. The variance of the new method was substantially reduced by employing multiple trials to improve the estimate of autocorrelation with non-zero time lags. The new method was then validated using a double inverted pendulum model stabilized by two different state-feedback controllers and perturbed by internal noise, a reasonable model of human balancing which can describe the widely-reported ‘ankle’ and ‘hip’ strategies [28]. In particular, compared to the ordinary least squares method, the controller gain identified by the new method was considerably more accurate, yielding errors of $\sim$10% or less. The numerical simulation examples indicate that the new method can be used to identify human postural dynamics from experimental data. Given a biomechanically plausible model of the relevant gravito-inertial mechanics, the net multi-joint impedance, whether due to intrinsic mechanics or feedback control, may also be identified.

### Caveats of parametric model fitting

Like other parametric system identification techniques, the new method relies heavily on the model which determines the structure of ${\mathbf {A}}$ and ${\mathbf {B}}$. It should also be noted that $\mathbf {K_x}$ identified from the method is the gain matrix of a linear full-state feedback controller. Consequently, several trade-offs must be considered when developing models and interpreting results. As presented in this study, one may model a human as a double inverted pendulum with joint positions and velocities as its states and joint torques as its control input. With this model, the gain ${\mathbf {K}}_{\mathbf {x}}$ should be interpreted as the apparent impedance seen at the ankle and the hip, i.e., stiffness and damping at each joint as well as coupling between them. Depending on the order of the model and the physical meaning of the state and input vectors, the precise meaning of ${\mathbf {K}}_{\mathbf {x}}$ will vary. Therefore, the state vector and the model order should be carefully determined, based on the purpose of modeling. Moreover, the method does not draw any conclusions about underlying neural processes but only their products; it only identifies the net contributions from all control components such as intrinsic mechanical impedance and neural feedback control.

Significant time delay due to limited neural signal transmission rate is another important factor that makes human motor control challenging. However, the current work did not incorporate this aspect of human postural control. To identify time delay in the system, more sophisticated methods are required. In recent literature, the limitation of neural transmission has often been modeled as a pure time-delay in state feedback control [4, 6, 29]. This would essentially increase the order (or the maximum lag) of the model (12). Neglecting measurement noise, that model is equivalent to the widely studied auto-regressive models with order larger than one, and there exist a number of papers treating such models with scalar [30] and multi-dimensional state variables [20]. Both the unknown model order (equivalent to the unknown time delay) and the model parameters can be estimated, as briefly presented in Appendix 2: Yule-Walker equations for multi-variate autoregressive models. Augmenting the present methods with such features is left for future work.

### Important assumptions

The new method requires a number of modeling assumptions including 1) the stochastic dynamics of human balancing is linear and time-invariant (stationary), 2) the number of independent measurements equals the order of the system (hence ${\mathbf {H}}^{-1}$ exists), and 3) the process and measurement noises are white and mutually uncorrelated.

#### Linear and stationary processes

A mechanical system with any controller (nonlinear, discontinuous, or higher order) must yield at least the lower-order behavior modeled here. Musculo-skeletal mechanics acts to smooth out discontinuities. The remaining nonlinearities would either be differentiable or resemble noise, and small motions would justify a linearized representation. Indeed it has been widely reported that unperturbed human balancing exhibits only subtle movement [13, 27].

The stationarity of human balance is debatable [5, 31, 32]; due to fatigue or change in control strategy (e.g., transitioning between an ‘ankle strategy’ and a ‘hip strategy’ [28]) during balancing, the system may exhibit time-varying dynamics. Stationarity should be established before applying the new method to identify human postural control.

#### Existence of H

Whether ${\mathbf {H}}^\mathbf {{-1}}$ exists or not depends on the model. If one develops a joint-level human balance model, joint angular positions and velocities can be measured with reasonably high accuracy with available technologies, e.g., motion capture systems (MOCAP), inertial measurement units (IMUs), or goniometers. In general it becomes harder to obtain full measurement of states as more complex features of postural control are included in the model (e.g., muscle dynamics or neural time delay). On the other hand, [17–19] have shown that an appropriate system order and parameters may be identified from partial measurements for single-input systems. Further investigation and application of such methods to the analysis of human postural control is left for future work.

#### White and mutually uncorrelated noise

The new method relies heavily on the assumption that all noise processes in (10) are white and uncorrelated with each other. However, some studies have indicated that biological noise may best be described by ‘pink’ noise or Brownian noise [33]. Moreover, linear models lump all the higher-order and nonlinear terms of a real human system into process noise, which might not be white. However, it should be noted that the purpose of system identification is to parameterize a model which may provide mechanically feasible explanations of observations and guide experiments to test hypotheses. In that sense, any model is wrong, and white noise may be wrong, but it is a convenient and useful approximation.

### Strength of the new method compared to the ordinary least squares method

We used a scalar stochastic dynamic system to analyze properties of the new method. In Fig. 1, it was shown that the variance and bias of the new method are sensitive to the size of the true system parameter. The method’s performance degraded when *a* was close to 0 (in the 1D model). In the multi-joint model, it would correspond to the case when $\Vert {\mathbf {A}}_{\mathrm{cl}}\Vert$ is close to 0. However, such a case is quite rare in biological systems. In particular, $\Vert {\mathbf {A}}_{\mathrm{cl}}\Vert =0$ implies that the neural controller rejects any perturbation within one sampling interval.

It was also shown that the quality of the estimate is sensitive to the size of measurement noise, or more precisely, the size of measurement noise relative to process noise (internal biological noise), $\sigma _r$. Both the ordinary least squares method and the new method performed better as measurement noise decreased. When $\sigma _r=0$, the ordinary least squares method provided a very accurate estimate of the system parameter *a* as shown in Fig. 1 and it outperformed the new method. However, as $\sigma _r$ became larger, error in the ordinary least squares method increased rapidly. In contrast, the new method showed consistent performance across a range of $\sigma _r$ values. Moreover, recruiting multiple auto-correlation matrices with different time lags ($m=10$) substantially improved the precision of the new method and provided accurate estimates for values of $\Vert a \Vert \sim 0$. The improvement can easily be extended to the multi-dimensional case as it does not require any difficult operations. The performance difference between the new method and the conventional ordinary least squares method was even more pronounced in the double inverted pendulum example as shown in Figs. 4 and  5.

Furthermore, with the known parameters ${\mathbf {A}}$ and ${\mathbf {B}}$ based on the gravito-inertial model, the controller gain matrix could be estimated. The mean error of estimation of controller gain obtained from the new method was much smaller than that from the ordinary least squares method (Figs. 4 and 5), especially when measurement noise was large. Sensitivity to measurement noise is an important practical consideration. It has been reported that the variability of joint angles during quiet standing is on the order of 0.1 deg [6, 27]. The measurement errors of state-of-the-art IMUs, 0.2 - 0.3 deg, [34] is comparable to and perhaps larger than sway motion during quiet standing. This paper showed that when measurement noise was comparable to process noise, the ordinary least squares method can be substantially biased, while our method was unbiased for even larger noises.

The practical implication is quite striking. While measurement noise can be further reduced by setting up high-precision MOCAP in the lab, such high-precision measurement systems are usually expensive and require large space. If clinicians are to diagnose patients remotely in at-home settings, they may not have access to accurate measurement systems (e.g., MOCAP or high-precision IMUs). In that case, our method would be an effective alternative to the conventional ordinary least squares method because it does not require such high-precision sensors.

### Wider application

The method proposed in this paper is applicable to any linear, discrete-time stochastic system, thus relevant to a broad range of human system studies. For example, the new method appears to be applicable to the study of rhythmic movements, another important field in human motor control [35, 36]. For example, it is possible to quantify the degree of stability of walking [15] or rhythmic arm movement [37]. The relevance of the proposed framework to rhythmic movement is detailed in Appendix 5: Stability assessment of rhythmic movement. In a recent study, Ahn and Hogan [15] have shown how to obtain accurate assessment of gait stability by correcting the bias due to the short duration of experimental time series. However, that method was limited to a scalar human walking model and not easily extensible to the multi-joint models which are typical of human systems. Moreover, significant error in human motion measurement systems was not accounted for. Combining the strength of the new method with the results of Ahn and Hogan [15] may improve the state-of-the-art in stability assessment of human walking [38]. The same technique may also improve experimental stability assessment of legged robots.

Another interesting field of application is motor learning [16, 39]. In motor learning studies, how humans learn a task from observing errors in each trial is often modeled as a linear discrete-time system with some feedback mechanism as in (10). Typical human motor learning models assume measurement noise and process noise are the same ($\mathbf {v=w}$ in (1)). Due to this assumption, the least squares estimate requires additional correction as shown in [16] while our new method can readily be applied. Recent studies [16, 39] have examined a scalar dynamic model which assumes that a task error can be represented by a scalar variable. A method for multi-dimensional systems, as presented in the current study, would enable studies of how humans learn complex tasks in which error cannot be simply represented by a single number.

## Conclusion

This study presented a mathematically rigorous system identification method for identifying dynamics of unperturbed balance. With a biomechanically reasonable model of the multi-joint human body, the gains of a state-feedback controller can also be estimated without any information about the system’s noise strength. A numerical example with a double inverted pendulum model of human quiet standing validated the method.

Methods to assess human motor control have significant practical importance. They may allow quantitative diagnosis of individual patients and development of customized treatment plans. With an aging population, technology-assisted human mobility is a growing need. The methods presented here may allow better assessment of technology-assisted mobility, which may eventually lead to development of customized assistive and / or rehabilitative technologies.

## Data Availability

Not applicable.

## Appendices

### Appendix 1: Ordinary least squares

For the zero-mean discrete timeseries {${\mathbf {z}}_t$}$_1^N$ obtained from the system (1),

$$\begin{aligned} {\mathbf {z}}_{t+1}&= \mathbf {Hx}_{t+1} + {\mathbf {v}}_{t+1} \\&= \mathbf {H(Ax}_t+\mathbf {Gw}_t)+{\mathbf {v}}_{t+1} \\&=\mathbf {HAH}^{-1}({\mathbf {z}}_t-{\mathbf {v}}_t)+\mathbf {HGw}_t+{\mathbf {v}}_{t+1}\\&=\mathbf {HAH}^{-1}{\mathbf {z}}_t-\mathbf {HAH}^{-1}{\mathbf {v}}_t+\mathbf {HGw}_t+{\mathbf {v}}_{t+1}, \end{aligned}$$

one can form the over-determined system

$$\begin{aligned} \underbrace{ \begin{pmatrix} {\mathbf {z}}_2^T\\ {\mathbf {z}}_3^T\\ \vdots \\ {\mathbf {z}}_N^T\\ \end{pmatrix} }_{{\mathbf {T}}} = \underbrace{ \begin{pmatrix} {\mathbf {z}}_1^T\\ {\mathbf {z}}_2^T\\ \vdots \\ {\mathbf {z}}_{N-1}^T\\ \end{pmatrix} }_{{\mathbf {W}}} \underbrace{(\mathbf {HAH}^\mathbf {-1})^T}_{{\varvec{\Phi }}} \end{aligned}$$

or succinctly

$$\begin{aligned} {\mathbf {T}}={\mathbf {W}}{\varvec{\Phi }} \end{aligned}$$

which can be readily solved using the usual least-squares estimator

$$\begin{aligned} {\hat{\varvec{\Phi }}}_{\mathrm{OLS}}&= ({\mathbf {W}}^T{\mathbf {W}})^{-1}{\mathbf {W}}^T{\mathbf {T}}\\&= ({\mathbf {z}}_1{\mathbf {z}}_1^T+\cdots + {\mathbf {z}}_{N-1}{\mathbf {z}}_{N-1}^T)^{-1}\\&\times ({\mathbf {z}}_1{\mathbf {z}}_2^T+\cdots + {\mathbf {z}}_{N-1}{\mathbf {z}}_{N}^T)\\&= {\hat{\mathbf {R}}}_\mathbf {zz}(0)^{-1}{\hat{\mathbf {R}}}_\mathbf {zz}(1)\\&= ({\mathbf {H}}{\hat{\mathbf {A}}}_{\mathrm{OLS}}{\mathbf {H}}^{-1})^T \end{aligned}$$

Rearranging, the ordinary least squares estimate is obtained as

21 $$\begin{aligned} \hat{{\mathbf {A}}}_{\mathrm{OLS}} = {\mathbf {H}}^{-1}{\hat{\mathbf {R}}}_\mathbf {zz}(1){\hat{\mathbf {R}}}_\mathbf {zz}(0)^{-1}{\mathbf {H}} \end{aligned}$$

which is equivalent to (8) with $k=0$.

### Appendix 2: Yule-Walker equations for multi-variate autoregressive models

If one assumes a zero-mean discrete timeseries {${\mathbf {z}}_t$}$_1^N$ is an autoregressive process, a method to estimate the appropriate order *p* of the model

$$\begin{aligned} {\mathbf {z}}_{t} = {\mathbf {A}}_1 {\mathbf {z}}_{t-1} + {\mathbf {A}}_2 {\mathbf {z}}_{t-2} \cdots +{\mathbf {A}}_p {\mathbf {z}}_{t-p} + {\mathbf {e}}(t) \end{aligned}$$

and the corresponding coefficients ${\mathbf {A}}_j$ can be established. By multiplying ${\mathbf {z}}_{t-k}^T$ to each side of equation, taking expectation, and noting that $E\{ {\mathbf {e}}(t){\mathbf {z}}(t-k)^T \} = {\mathbf {0}}$, one can obtain

$$\begin{aligned} {\mathbf {R}}_{\mathbf {zz}}(k)&= {\mathbf {A}}_1{\mathbf {R}}_{\mathbf {zz}}(k-1) + {\mathbf {A}}_2{\mathbf {R}}_{\mathbf {zz}}(k-2) + \cdots \\&+ {\mathbf {A}}_{p}{\mathbf {R}}_{\mathbf {zz}}(k-p), \forall k > 0 \end{aligned}$$

Substituting $k=1,2,\cdots ,p$ in the above equation, with ${\mathbf {R}}_{\mathbf {zz}}(k)^T={\mathbf {R}}_{\mathbf {zz}}(-k)$, one can obtain the set of equations referred to as the Yule-Walker equations [20, 30]:

$$\begin{aligned} {\mathbf {R}}_{\mathbf {zz}}(1)&= {\mathbf {A}}_1{\mathbf {R}}_{\mathbf {zz}}(0) + {\mathbf {A}}_2{\mathbf {R}}_{\mathbf {zz}}^T(1) + \cdots \\&+ {\mathbf {A}}_{p}{\mathbf {R}}_{\mathbf {zz}}^T(p-1) \\ {\mathbf {R}}_{\mathbf {zz}}(2)&= {\mathbf {A}}_1{\mathbf {R}}_{\mathbf {zz}}(1) + {\mathbf {A}}_2{\mathbf {R}}_{\mathbf {zz}}(0) + \cdots \\&+ {\mathbf {A}}_{p}{\mathbf {R}}_{\mathbf {zz}}^T(p-2) \\&\vdots \\ {\mathbf {R}}_{\mathbf {zz}}(p)&= {\mathbf {A}}_1{\mathbf {R}}_{\mathbf {zz}}(p-1) + {\mathbf {A}}_2{\mathbf {R}}_{\mathbf {zz}}(p-2) + \cdots \\&+ {\mathbf {A}}_{p}{\mathbf {R}}_{\mathbf {zz}}(0) \\ \end{aligned}$$

which can also be written as

$$\begin{aligned}&\underbrace{ \begin{pmatrix} {\mathbf {R}}_{\mathbf {zz}}(1), {\mathbf {R}}_{\mathbf {zz}}(2), \cdots , {\mathbf {R}}_{\mathbf {zz}}(p) \end{pmatrix} }_{\tilde{{\mathbf {r}}}} \\&= \underbrace{ \begin{pmatrix} {\mathbf {A}}_1, {\mathbf {A}}_2, \cdots , {\mathbf {A}}_p \end{pmatrix} }_{{\varvec{\Phi }}}\times \\&\underbrace{ \begin{pmatrix} {\mathbf {R}}_{\mathbf {zz}}(0) &{} {\mathbf {R}}_{\mathbf {zz}}(1) &{} \cdots &{} {\mathbf {R}}_{\mathbf {zz}}(p-1) \\ {\mathbf {R}}_{\mathbf {zz}}^T(1) &{} {\mathbf {R}}_{\mathbf {zz}}(0) &{} \cdots &{} {\mathbf {R}}_{\mathbf {zz}}(p-2) \\ \vdots \\ {\mathbf {R}}_{\mathbf {zz}}^T(p-1) &{} {\mathbf {R}}_{\mathbf {zz}}(p-2) &{} \cdots &{} {\mathbf {R}}_{\mathbf {zz}}(0) \\ \end{pmatrix} }_{\tilde{{\mathbf {R}}}} \end{aligned}$$

or succinctly

$$\begin{aligned} \tilde{{\mathbf {r}}}=\Phi {\tilde{R}} \end{aligned}$$

Note that this is a well-posed system with the same number of equations as unknowns. The matrix ${\tilde{\mathbf {R}}}$ is full-rank and symmetric, so that invertibility is guaranteed. Therefore the coefficients or the system parameters ${\varvec{\Phi }}$ can be estimated by

$$\begin{aligned} {\hat{\varvec{\Phi }}}={{\tilde{\mathbf {r}}}}{\tilde{\mathbf {R}}}^{-1} \end{aligned}$$

There are various ways to determine the order of the system *p*. For example, the proper order *p* can be determined by minimizing the Akaike information criterion (AIC). Readers are referred to [20] for more details. Note that for the model with order $p=1$, the resultant parameter estimate of ${\hat{\mathbf {A}}}_1$ is the same as the ordinary least squares method, i.e., ${\hat{\mathbf {A}}}_1 = {\mathbf {R}}_{\mathbf {zz}}(1){\mathbf {R}}_{\mathbf {zz}}(0)^{-1}$

### Appendix 3: Double inverted pendulum linearization

The equations of motion of the double inverted pendulum model are

22 $$\begin{aligned} {\mathbf {M}}({\mathbf {q}})\mathbf {\ddot{q}} + {\mathbf {C}}({\mathbf {q}},\mathbf {{\dot{q}}})\mathbf {{\dot{q}}} + {\mathbf {G}}({\mathbf {q}}) = \varvec{\tau } = {\mathbf {u}}+\varvec{\eta }, \end{aligned}$$

where ${\mathbf {M}}({\mathbf {q}}) \in \mathbb {R}^{2\times 2}$ is the inertia matrix, ${\mathbf {C}}({\mathbf {q}},\mathbf {{\dot{q}}}) \in \mathbb {R}^{2\times 1}$ are the Coriolis and centrifugal terms, ${\mathbf {G}}({\mathbf {q}}) \in \mathbb {R}^{2\times 1}$ are the gravitational torques, and $\varvec{\tau } = [\tau _1, \tau _2]^T \in \mathbb {R}^{2\times 1}$ is the joint torque vector, which is sum of control input ${\mathbf {u}}$ and motor noise $\varvec{\eta }$. The generalized coordinates are ${\mathbf {q}} = [q_1, q_2]^T \in \mathbb {R}^{2\times 1}$, where $q_1$ is the angle of the lower body link (link 1) measured from the upright position, and $q_2$ is the relative angle of the upper body link (link 2) measured from the lower body link position. The angles represent sagittal plane ankle and the hip joint motions, respectively.

Defining the state variables as ${\mathbf {x}} = [{\mathbf {q}}^T, {\dot{\mathbf {q}}}^T]^T$, (22) can be rewritten as

$$\begin{aligned} {\dot{\mathbf {x}}} = \begin{bmatrix} {\dot{\mathbf {q}}}\\ \mathbf {-M(q)}^{-1}(\mathbf {C}(\mathbf {q},{\dot{\mathbf {q}}})\dot{\mathbf {q}}+\mathbf {G(q)})+\varvec{\tau } \end{bmatrix}. \end{aligned}$$

The nonlinear equations of motion can be linearized about the equilibrium point of the model or the upright balancing posture (${\mathbf {x}}_* = {\mathbf {0}}$, ${\mathbf {u}}_* = {\mathbf {0}}$, and $\varvec{\eta }_*={\mathbf {0}}$) as follows

$$\begin{aligned} \dot{{\mathbf {x}}} = \mathbf {A_c}{\mathbf {x}} + {\mathbf {B}}_{\mathbf {c}}{\mathbf {u}}+{\mathbf {B}}_{\mathbf {c}}\varvec{\eta } \end{aligned}$$

where ${\mathbf {A}}_{\mathbf {c}}$ and ${\mathbf {B}}_{\mathbf {c}}$ are linearized state and input matrices, respectively. Subscript *c* stands for continuous-time. Evaluation of the Taylor expansion around a fixed point yields the following, very simple equations, given in block form by:

$$\begin{aligned} {\mathbf{A}}_{{\mathbf{c}}} & = \left[ {\begin{array}{*{20}l} {\mathbf{0}} & {\mathbf{I}} \\ { - {\mathbf{M}}^{{ - 1}} \frac{\partial }{{\partial {\mathbf{q}}}}{\mathbf{G}}} & {\mathbf{0}} \\ \end{array} } \right]_{{{\mathbf{x}} = {\mathbf{x}}_{*} ,\varvec{\tau } = \varvec{\tau } _{*} }} \\ {\mathbf{B}}_{{\mathbf{c}}} & = \left[ {\begin{array}{*{20}l} {\mathbf{0}} \\ {{\mathbf{M}}^{{ - 1}} } \\ \end{array} } \right]_{{{\mathbf{x}} = {\mathbf{x}}_{*} ,\varvec{\tau } = \varvec{\tau } _{*} }} \\ \end{aligned}$$

With the state feedback controller $\mathbf {u=-K_x}\mathbf {x}$, the closed-loop system matrix is obtained as

$$\begin{aligned} {\mathbf {A}}_{cl}={\mathbf {A}}-\mathbf {BK}_{\mathbf {x}}. \end{aligned}$$

### Appendix 4: Discrete-to-continuous conversion

The method described in this paper is based on discrete-time system model, but sometimes continuous-time models are more convenient. In such cases, the discrete-time system parameters obtained using the new method should be properly converted to continuous-time approximation.

In the example in Numerical simulation: balance model, we used semi-implicit Euler integrator to integrate forward dynamics. Therefore the conversion from the continuous-time system parameters ${\mathbf {A}}_{\mathrm{c}}$ to its discrete-time counterpart ${\mathbf {A}}_{\mathrm{d}}$ becomes

$$\begin{aligned} {\mathbf {A}}_{\mathrm{d}} = \begin{bmatrix} {\mathbf {I}} &{} dt{\mathbf {I}}\\ {\mathbf {0}} &{} {\mathbf {I}} \end{bmatrix} + \begin{bmatrix} {\mathbf {0}} &{} dt^2{\mathbf {I}}\\ {\mathbf {0}} &{} dt{\mathbf {I}} \end{bmatrix} {\mathbf {A}}_{\mathrm{c}} \end{aligned}$$

where *dt* is the sample time interval. If we assume that the top rows of ${\mathbf {A}}_{\mathrm{c}}$ are $[\mathbf {0, I}]$,

$$\begin{aligned} {\mathbf {A}}_{\mathrm{d}} = \begin{bmatrix} {\mathbf {I}} &{} {\mathbf {0}}\\ {\mathbf {0}} &{} {\mathbf {I}} \end{bmatrix} + \begin{bmatrix} dt{\mathbf {I}} &{} dt^2{\mathbf {I}}\\ {\mathbf {0}} &{} dt{\mathbf {I}} \end{bmatrix} {\mathbf {A}}_{\mathrm{c}} \end{aligned}$$

therefore the discrete-time to continuous-time conversion is obtained as

$$\begin{aligned} {\mathbf {A}}_{\mathrm{c}} = \frac{1}{dt} \begin{bmatrix} {\mathbf {I}} &{} -dt{\mathbf {I}}\\ {\mathbf {0}} &{} {\mathbf {I}} \end{bmatrix} \left( {\mathbf {A}}_{\mathrm{d}} - \begin{bmatrix} {\mathbf {I}} &{} {\mathbf {0}}\\ {\mathbf {0}} &{} {\mathbf {I}} \end{bmatrix} \right) \end{aligned}$$

### Appendix 5: Stability assessment of rhythmic movement

Orbital stability of a limit cycle in state-space has one-to-one correspondence to the stability of a discrete return map, or Poincaré map. The eigenvalues of the linearized Poincaré map are called characteristic or Floquet multipliers [40, 41].

The Poincaré map ${\mathbf {x}} \mapsto P({\mathbf {x}})$ relates the state of a system after one cycle (${\mathbf {x}}_{t+1}$) and its current state (${\mathbf {x}}_{t}$): ${\mathbf {x}}_{t+1} = P({\mathbf {x}}_t)$. It follows that limit cycle trajectories correspond to the fixed point (${\mathbf {x}}^*$) of the map, ${\mathbf {x}}^* = P({\mathbf {x}}^*)$, and the (local) orbital stability of the limit cycle is equivalent to the stability of the corresponding fixed point of the map on the Poincaré section. To evaluate the effects of small perturbations on ${\mathbf {x}}^*$, the Poincaré map can be linearized:

$$\begin{aligned} {\mathbf {x}}_{t+1} - {\mathbf {x}}^* = \frac{\partial P}{ \partial {\mathbf {x}}}|_{(\mathbf {x=x}^*)} ({\mathbf {x}}_{t} - {\mathbf {x}}^*) \end{aligned}$$

Denoting ${\mathbf {A}}_{\mathbf {p}} = \frac{\partial P}{ \partial {\mathbf {x}}}|_{(\mathbf {x=x}^*)}$ and assuming ${\mathbf {x}}^*={\mathbf {0}}$ without loss of generality, also assuming white process and measurement noise, we obtain the following expression,

$$\begin{aligned} {\left\{ \begin{array}{ll} {\mathbf {x}}_{t+1} = {\mathbf {A}}_{\mathbf {p}} {\mathbf {x}}_{t} + \mathbf {Gw}_t\\ {\mathbf {z}}_{t} = {\mathbf {H}} {\mathbf {x}}_{t} + {\mathbf {v}}_t \end{array}\right. } \end{aligned}$$

to which (9) can be readily applied to obtain $\hat{{\mathbf {A}}}_{\mathbf {p}}$. The maximum Floquet multiplier is the eigenvalue of $\hat{{\mathbf {A}}}_{\mathbf {p}}$ with the largest modulus.

## Abbreviations

COM: Center of mass
COP: Center of pressure
OLS: Ordinary least squares
LQR: Linear quadratic regulator
MOCAP: Motion capture system
IMU: Inertial measurement unit
